# Adequate rectal preparation reduces hospital admission for urosepsis after transrectal ultrasound - guided prostate biopsy

**DOI:** 10.1590/S1677-5538.IBJU.2018.0181

**Published:** 2018

**Authors:** Yu-Chen Chen, Hao-Wei Chen, Shu-Pin Huang, Hsin-Chin Yeh, Ching-Chia Li

**Affiliations:** 1 Graduate Institute of Clinical Medicine, College of Medicine, Kaohsiung Medical University, Kaohsiung, Taiwan; 2 Department of Urology, Kaohsiung Medical University Hospital, Kaohsiung Medical University, Kaohsiung, Taiwan; 3 Department of Urology, Kaohsiung Municipal Ta-Tung Hospital, Kaohsiung, Taiwan

**Keywords:** Prostate, Prostatic Neoplasms, Ultrasound, High-Intensity Focused, Transrectal

## Abstract

**Objectives:**

Previous studies have compared infectious outcomes on the basis of whether rectal preparation was performed; however, they failed to evaluate the quality of each rectal preparation, which may have led to confounding results. This study aimed to compare hospitalizations for urosepsis within 1 month after transrectal ultrasound-guided prostate biopsy between patients with adequate and traditional rectal preparations.

**Materials and Methods:**

Between January 2011 and December 2016, a total of 510 patients who underwent transrectal ultrasound - guided prostate biopsy at our institutions and were orally administered prophylactic antibiotics (levofloxacin) were included. Two rectal preparations were performed: (1) adequate rectal preparation confirmed by digital rectal examination and transrectal ultrasound (Group A, n = 310) and (2) traditional rectal preparation (Group B, n = 200). All patient characteristics were recorded. A logistic regression model was used to assess the effects of the two different rectal preparations on urosepsis, adjusted by patient characteristics.

**Results:**

There were a total of three and nine hospitalizations for urosepsis in Groups A and B, respectively. Differences in the demographic data between the two groups were insignificant. Logistic regression showed that adequate rectal preparation before biopsy significantly decreased the risk for urosepsis after biopsy (adjusted odds ratio: 0.2; 95% confidence interval: 0.05 – 0.78; P = 0.021).

**Conclusions:**

Adequate rectal preparation could significantly reduce hospitalizations for urosepsis within 1 month after transrectal ultrasound-guided prostate biopsy. The quality of rectal preparation should be evaluated before biopsy. If adequate rectal preparation is not achieved, postponing the biopsy and adjusting the rectal preparation regimen are suggested.

## INTRODUCTION

Transrectal ultrasound - guided prostate biopsy (TRUS - Bx) is a standard procedure used to diagnose prostate cancer. Although it is generally considered safe, infectious complications related to TRUS - Bx occur, which include urinary tract infection (UTI; > 6%), prostatitis, and sepsis, requiring hospital admission for intravenous antibiotic treatment (~3%) (1, 2). It is believed that bacteria in the rectum are seeded in the prostate, bladder, and / or bloodstream by the hollow core biopsy needle traversing the rectum into the prostate during TRUS - Bx and may lead to post - biopsy infection (3). The effect of rectal preparation on reducing post - TRUS - Bx infections has been debated in previous studies and remains controversial in the American Urological Association and European Association of Urology Nurses guidelines (2, 4). Although previous studies have reported on whether rectal preparation was conducted (5, 6) and whether different rectal preparation regimens influenced the post - biopsy infectious complication rates (7), they failed to confirm the quality of rectal preparation, which may have led to confounding results. In fact, an inadequate rectal preparation occurs frequently despite using cleansing enema. Hence, we focused on the quality of rectal preparations, and this study aimed to evaluate whether achieving adequate rectal preparations may be effective in reducing hospitalizations for urosepsis within 1 month after TRUS - Bx.

## MATERIALS AND METHODS

Between January 2011 and December 2016, all patients who underwent TRUS - Bx at our medical centers were retrospectively reviewed. Demographic data, such as age, prostate - specific antigen (PSA) levels, prostate volumes, body mass index (BMI), diabetes mellitus (DM), hypertension (HTN), and TRUS - Bx pathological reports, were analyzed. Hospitalizations for urosepsis within 1 month after TRUS - Bx were assessed via a chart review. This study was approved by the institutional review board of Kaohsiung Medical University Hospital (ID: KMUHIRB-E(I)-20170227).

### Inclusion / Exclusion Criteria

The indications for biopsy were high serum PSA levels or abnormal findings on digital rectal examination (DRE), TRUS, or magnetic resonance imaging, from which prostate cancer was strongly suspected. Patients who underwent TRUS - Bx between January 2011 and December 2016 and received prophylactic antibiotics (500 mg of levofloxacin) orally once a day starting on the day of the biopsy and lasting for 2 days were included. The exclusion criteria were UTI, acute bacterial prostatitis (NIH classification I), chronic bacterial prostatitis (NIH classification II), or use of quinolone for some other reason within 3 months before the prostate biopsy. Patients who were lost to follow-up and those with unclear medical and procedure reports were also excluded.

### Study Protocol

All biopsies within the abovementioned period were scheduled as inpatient procedures. All patients received a phosphate enema before the TRUS - Bx. Two rectal preparations were performed: (1) adequate rectal preparation confirmed by DRE and TRUS (Group A) and (2) traditional rectal preparation (Group B). In Group A, DRE and TRUS were performed immediately before the TRUS - Bx to confirm the quality of the rectal preparation and to ensure that each patient achieved an adequate rectal preparation. We defined an adequate rectal preparation as the achievement of an empty rectal vault, which meant that there was no gross stool on the gloved finger during DRE nor was it visualized in the rectal vault under TRUS. The TRUS - Bx was cancelled if an adequate rectal preparation was not achieved and re - arranged until no stool was found under DRE and TRUS. For those who did not achieve an adequate rectal preparation, an additional bowel movement was required, and / or an intensified rectal preparation regimen was considered depending on the patient's clinical condition. In Group B, the rectal preparation quality was not evaluated, and the TRUS - Bx was performed as scheduled, even if the rectal vault was not completely empty. Thus, we identified Group B as the traditional rectal preparation group.

All patients in both groups received the same prophylactic antibiotics (500 mg of levofloxacin) orally, once a day, starting on the day of the biopsy and lasting for 2 days. After providing adequate information of the procedure and on the potential hazards and obtaining the informed consent from patients, TRUS - Bx was performed in an operating room with local anesthesia. For all patients, the rectal wall was cleansed using povidone - iodine before the TRUS - Bx. In the lithotomy position, an 18 - gauge Bard^®^ Max - Core^®^ Disposable Core Biopsy needle was used to obtain the biopsy cores. A total of 12 cores were collected from the prostate of all patients, that is, six cores from each side.

The patients were discharged once they achieved smooth micturition after the biopsy. All patients returned to the urology outpatient clinic 1 week after the biopsy to receive their pathology report, and we could ascertain whether any infectious or noninfectious complications had occurred. The patients were also instructed to return to the hospital if they developed any symptoms of infection.

### Outcome

The study's end - point was hospitalization for urosepsis within 1 month after the TRUS - Bx. Sepsis was defined as the presence of two or more of the following conditions along with bacterial infection: temperature of > 38.0°C or < 36.0°C, heart rate > 90 bpm, respiratory rate > 20 breaths / min or respiratory alkalosis, and white blood cell count > 12.000 or immature cell form count > 10% in proportion (8). For the patients with urosepsis, both urine and blood samples were collected for culture and fully evaluated.

### Statistical analyses

Fisher's exact and Mann - Whitney U- -test were used for categorical and continuous variables, respectively. Multivariable logistic regression analysis was used to assess the effects of the two different rectal preparations (adequate rectal preparation versus traditional rectal preparation) on the occurrence of urosepsis within 1 month after TRUS - Bx. Other potential factors considered were the two different prophylactic antibiotic protocols, age, BMI, DM, HTN, prostate volume, and biopsy pathological results as covariates.

All tests were two - sided, and P-values ≤ 0.05 were considered statistically significant. Analyses were performed using IBM SPSS Statistics version 22.0 (IBM^®^ Corporation, NY, USA).

## RESULTS

A total of 510 patients who underwent TRUS - Bx in Group A (n = 310) and Group B (n = 200) met the inclusion criteria. Differences in the patients’ mean age, BMI, PSA level, prostate volume, incidence of DM and HTN, and biopsy pathological results between the two groups were not significant (Table-1). Multiple logistic regression showed that only patients who achieved an adequate rectal preparation before TRUS - Bx had a decreased risk of developing urosepsis after TRUS - Bx (adjusted odds ratio: 0.2; 95% confidence interval: 0.05 – 0.78, P = 0.021) (Table-2).

**Table 1:** Demographic data of the 510 patients stratified in accordance with adequate rectal preparation (Group A) and traditional rectal preparation (Group B).

Variables		Group A (n = 310)	Group B (n = 200)	P-value
Mean age, y (range)		68.75 (46-86)	69.91 (45-86)	0.072
Mean BMI, kg/m^2^ (range)		25.11 (16.4-43.6)	24.57 (14.3-33.1)	0.084
DM, n (%)		72 (23)	41 (21)	0.5
HTN, n (%)		143 (46)	95 (48)	0.8
Mean PSA, ng/dL (range)		22.65 (4.1-386)	31.37 (2.15-492)	0.3
Mean prostate volume, mL (range)		52.1 (16.7-197)	49.2 (10.5-181)	0.065
**Biopsy pathological result, n (%)**				0.6
	No prostate cancer	212 (68)	139 (70)	
	Gleason score < 7	22 (7)	19 (9)	
	Gleason score 7	25 (8)	11 (6)	
	Gleason score > 7	51 (17)	31 (16)	

BMI, body mass index; DM, diabetes mellitus; HTN, hypertension; PSA, prostate-specific antigen

**Table 2:** Logistic regression of potential factors on hospital admissions for urosepsis.

Variables		Hospitalizations for urosepsis	
		Adjusted OR (95% CI)	P-value
Age		0.95 (0.9-1.02)	0.2
BMI		0.99 (0.82-1.2)	0.9
DM		0.77 (0.15-3.98)	0.8
HTN		1.17 (0.34-4.06)	0.8
Prostate volume		1.01 (0.98-1.03)	0.9
PSA		1.01 (0.99-1.01)	0.7
**Biopsy pathological results**			
	No prostate cancer	Ref.	
	Prostate cancer Gleason score < 7	2.7 (0.49-15.04)	0.3
	Prostate cancer Gleason score 7	2.41 (0.23-25.19)	0.5
	Prostate cancer Gleason score > 7	2.47 (0.43-14.03)	0.3
**Rectal preparation**			
	Traditional rectal preparation	Ref.	
	Adequate rectal preparation	0.2 (0.05-0.78)	0.021

**BMI =** body mass index; **CI =** confidence interval; **DM =** diabetes mellitus; **HTN =** hypertension; **OR =** odds ratio; **PSA =** prostate-specific antigen

There were three and nine cases of urosepsis in Group A and Group B, respectively. Culture data were obtained for all infection - related hospitalizations (Table-3). The cultures revealed presence of Escherichia coli in two and five cases in Group A and Group B, respectively. Fluoroquinolone - resistant organisms were identified in two and four cases in Group A and Group B, respectively. No statistically significant differences were identified in the positive culture findings, rate of Escherichia coli positivity, and rate of quinolone resistance between the two groups.

**Table 3:** Culture data of the patients with infection-related hospitalizations in the two groups.

Variables	Group A (n = 310)	Group B (n = 200)	P-value
Infectious number, n	3	9	
Positive culture finding, n (%)	2 (67)	5 (56)	0.7
*Escherichia coli* rate, %†	100 (2/2)	60 (3/5)	0.3
Quinolone resistance rate, %‡	100 (2/2)	80 (4/5)	0.5

† The *Escherichia coli* rate was defined as the number of positive *Escherichia coli* findings among the positive culture findings.

‡ The quinolone resistance rate was defined as the number of organisms resistant to quinolone among the positive culture findings.

## DISCUSSION

Prostate biopsy is a well - established procedure used to diagnose prostate cancer and can be performed transrectally or transperineally. TRUS - Bx is the most common method utilized because of the need for local anesthesia only; however, it is accompanied by an infectious complication rate of 0.1 – 7.0%, including UTI, prostatitis, epididymitis, orchitis, bacteremia, and urosepsis, requiring hospital admission for treatment with intravenous antibiotics (1, 2, 9). The transperineal approach, which was developed to reduce infection by avoiding the rectum, leads to generally low rates of infectious complications (10, 11). However, this approach is less commonly used because of the need for general anesthesia, greater amount of pain involved, and higher potential risk of perineal hematoma (12). Therefore, it is essential to place a renewed focus on strategies to reduce infectious complications after TRUS - Bx.

The causative bacteria for post - biopsy infection are seeded into the prostate, bladder, and / or bloodstream by the hollow core biopsy needle traversing the rectum into the prostate / bladder during TRUS - Bx (3). Therefore, rectal preparation before TRUS - Bx could theoretically prevent post - biopsy infection by reducing the rectal bacterial load associated with feces and thereby reducing the bacteria brought into the prostate and bladder. However, the effects of rectal preparation on reducing post - TRUS - Bx infections remain controversial. A Cochrane review found that enemas with antibiotics were associated with fewer instances of bacteremia (a reduction from 28% to 4%), but there was no difference in the occurrence of bacteriuria or fever (13). Some reports have suggested that rectal preparations, such as enemas or bisacodyl administration, decrease the rate of infectious complications (5, 7), whereas other studies have suggested otherwise (14). Because of the lack of evidence, rectal preparation has remained controversial in the 2011 European Association of Urology Nurses guidelines and the updated 2016 American Urological Association white paper (2, 4), although almost all patients (79 – 81%) undergo rectal preparation before biopsy in daily practice (15).

Similar to an inadequate bowel preparation reportedly occurring in up to 25% of colonoscopies in the USA (16), we also found that an inadequate rectal preparation occurs frequently (gross stool was often found on DRE or TRUS before TRUS - Bx), especially in those with chronic constipation or those noncompliant to the rectal preparation regimen. Therefore, unlike previous studies (5-7, 13), we aimed to document the quality of rectal preparations, and our results revealed that an adequate rectal preparation could significantly decrease the post - TRUS - Bx infection rate. Moreover, because of different responses in patients even under the same rectal preparation regimen, the quality of rectal preparation should be evaluated before TRUS - Bx, and an intensified rectal preparation regimen could be considered in patients with a history of an inadequate rectal preparation.

Clinically, hospitalization for urosepsis after TRUS - Bx has been estimated to cost 5.800 US dollars per event (17, 18), and it poses a potentially life-threatening risk to the patients and leads to mistrust in the doctor - patient relationship and subsequent patient transfer, leading to increased medical costs. In our study, the number of patients needing treatment was 28, which indicated that approximately 13.000 New Taiwan dollars of hospitalization with urosepsis could be saved upon 28 adequate rectal preparations.

Antimicrobial prophylaxis is recommended for all patients undergoing TRUS - Bx to defend against bacteria that are inevitably introduced from the rectum via the biopsy needle and reduce the risk of bacteriuria, bacteremia, and clinical infections after prostate biopsy (13). However, the increasing use of fluoroquinolones globally as prophylactic antibiotics has increased the overall resistance to fluoroquinolones, and infection after TRUS - Bx is most commonly caused by fluoroquinolone - resistant Escherichia coli (19, 20). Consistent with the findings of previous studies, Escherichia coli was the most common organism cultured among patients with urosepsis, and quinolone resistance rates were high in both groups in our study.

The limitations of our study are (1) its retrospective, nonrandomized design based on data derived from the medical records of the enrolled patients and the procedure notes of TRUS - Bx; (2) the study outcome which only included hospitalization for urosepsis within 1 month after the TRUS - Bx, but did not include other slightly infectious complications which only needed outpatient care. Further large prospective case - controlled studies are required to confirm the outcomes of the present study.

## CONCLUSIONS

On the basis of our results, adequate rectal preparation could significantly reduce hospitalizations for urosepsis after TRUS - Bx and avoid increased medical costs. The quality of rectal preparation should be evaluated before TRUS - Bx. We suggest that if adequate rectal preparation is not achieved, postponing the biopsy and adjusting the rectal preparation regimen are suggested.
